# COVID-19 Vaccination and Its Relation to New-Onset Diabetes: A Narrative Review

**DOI:** 10.7759/cureus.47056

**Published:** 2023-10-15

**Authors:** Deepa Rashmi Sivaprakasam, Harrison Obinna Ohiri, Mohammad S Asif, Muhammad Shahabul Jahangir, Muhammad Khubaib Gul Khan, Muhammad Asad Nabeel, Rai Muhammad Abdullah

**Affiliations:** 1 Medicine Department, Kapilan Healthcare, Chennai, IND; 2 Clinicals Department, United Memorial Medical Center, Houston, USA; 3 Medicine Department, University College of Medicine and Dentistry, Lahore, PAK

**Keywords:** severe acute respiratory syndrome coronavirus 2 (sars-cov-2), diabetic ketoacidosis, hyperglycemia, type 2 diabetes mellitus, type 1 diabetes mellitus, covid-19

## Abstract

The COVID-19 vaccination has been effective in preventing a lot of complications caused by SARS-CoV-2 and its variants. Meanwhile, diabetes mellitus, one of the root causes of many co-morbidities, exhibited itself during the COVID-19 pandemic and after COVID-19 vaccination. Diabetes mellitus introduced itself in a new perspective, leading to a variety of presentations and causing a significant number of emergency admissions. Many of the pre-diabetes patients with no prior history of diabetes developed fulminant type 1 diabetes mellitus (T1DM) after the COVID-19 vaccination. Some cases of conversion of type 2 diabetes mellitus (T2DM) into T1DM were reported. Some prediabetes/diabetes patients presented with the development of diabetic ketoacidosis after COVID-19 vaccination, whereas some previously healthy people with no relation to diabetes also developed acute exacerbations of new-onset T1DM or T2DM along with lethal ketoacidosis. The purpose of writing this review was to explore what kind of people are more prone to develop new-onset diabetes or diabetic complications, including diabetic ketoacidosis, the typical presentation of these patients, possible mechanisms that lead to these complications occurring after the COVID-19 vaccination, how they can be managed, and whether there is a good prognosis after management or not.

## Introduction and background

The outbreak of a respiratory disease started in December 2019 in China, leading to the discovery of the new highly contagious coronavirus SARS-CoV-2 after one month, with the most significant results through RT-PCR and molecular tests, and later on being announced as a pandemic by the WHO in March 2020 after a lot of cases with the same infection and pneumonia were reported [1-3].

Preexisting diabetes and new-onset diabetes due to the SARS coronavirus have been associated with high complication rates, poor outcomes, and mortality as an independent risk factor during severe acute respiratory syndrome coronavirus-1 (SARS-CoV-1) in 2003, with or without glucocorticoid treatment [4-6]. Like SARS-CoV-1, the emergence of COVID-19 has shown a bidirectional relationship with diabetes, i.e., diabetes leading to worsening of disease and COVID-19 leading to new-onset diabetes and hyperglycemia [7-9]. Patients with new-onset hyperglycemia and both diabetes types I and II with SARS-CoV infection showed poor outcomes, severe manifestations of coronavirus disease, all-cause deaths, higher complications (in new-onset/preexisting diabetes), and the development of insulin resistance, as compared to patients with preexisting diabetes or normal glycemic levels even without prior history of diabetes or dysglycemia [10-12]. Patients with COVID-19 admission presenting with impaired fasting glucose and diabetes showed high complications and vulnerable outcomes, along with worse signs and symptoms of dyspnea, fatigue, higher rates of ICU admissions, mechanical ventilation, or death [13-15].

COVID-19 was managed through medical treatment with drugs and symptom management, together with the management of diabetic emergencies, until the vaccine was developed [16,17]. More than 200 vaccines of different types (DNA-based, mRNA-based, live attenuated, inactivated, viral vector, subunit vaccine) were registered under clinical trials at the WHO in 2020 [18-20]. The implication of the vaccines has proven to show advantages in the prevention of COVID-19, but, on the other side, some side effects have been on the rise in different body systems [21-23].

This article was aimed at taking a review by searching databases through Scopus and PubMed on the effect of different types of vaccines on preexisting diabetes types I and II and hyperglycemia (developing after COVID-19 infection or preexisting before COVID-19 exposure) and observing whether vaccines lead to new-onset hyperglycemia and diabetes type I or II. Until now, there has been no data on the mechanism causing the complications of diabetes in vaccinated people. There is a need to explore the database to know the patterns and presentation of different types of diabetic complications after vaccination and how these complications occur and can be managed. The purpose of writing this review was to explore what kind of people are more prone to develop new-onset diabetes or diabetic complications, including diabetic ketoacidosis, the typical presentation of these patients, possible mechanisms that lead to these complications occurring after the COVID-19 vaccination, how they can be managed, and whether there is a good prognosis after management or not.

## Review

We searched databases from Scopus and PubMed and thoroughly reviewed almost all available studies (33) from January 2021 until 2023 in relation to COVID-19 vaccination and diabetes.

Vaccination and preexisting/new-onset type 2 diabetes mellitus (T2DM) and hyperglycemia

Abu-Rumaileh et al. reported a case of a 58-year-old African male in March 2021 who presented with a hyperosmolar hyperglycemic state (HHS) after receiving a Pfizer-BioNTech COVID-19 vaccine dose six days before. The patient was pre-diabetic with a mild hyperglycemia record evident from the blood glucose profile (the HbA1c record was not available) and a positive family history of T2DM in the patient’s mother and father. The patient presented with signs and symptoms of polyuria and dry mouth, along with altered mental status. His PCR analysis for COVID was negative, and he was admitted to the intensive care unit (ICU) and managed with 50 units of insulin glargine once daily until the achievement of glycemic control [24].

In May 2021, Mashira et al. reported the development of hyperglycemia in two males and one female with T2DM after receiving the COVISHEILD vaccine (Serum Institute of India Pvt. Ltd., Pune, India). All three patients were on oral agents for diabetes and had good glycemic control along with prevention. However, after receiving their first doses of COVISHEILD vaccines, the patients experienced increasing levels of glucose, but no adverse signs or symptoms occurred. The male patients achieved normal glucose levels within three and 15 days without any changes in interventions, but the female patient had to increase her metformin dose, and her hyperglycemia remained for one month. The authors pointed out vaccine-induced inflammation and immune response to be the probable causes behind hyperglycemia [25].

In July 2021, Edwards et al. reported three cases of obese male patients presenting with hyperglycemic crisis developing within one week after the adenovirus‐vectored COVID‐19 vaccine administration. Two of them were pre-diabetic with a history of hypertension, while the third person had only a history of diabetes with no positive family history of diabetes. Their blood profile showed low c-peptide levels and high HbA1c on admission, in accordance with hyperglycemia. They proposed the possibilities of the development of hyperglycemia in relation to SARS-CoV-2 infection, the effect of the virus on angiotensin-converting enzyme, and exposure to a vector-based vaccine. They concluded with the suggestion that the benefits of the vaccine still outweigh its adverse effects, so the patients should be screened for the development of hyperglycemia before the vaccination, and the results should be compared to prepare for future events of vaccination accordingly [26].

Lee et al. presented cases of rapid onset of HHS or diabetic ketoacidosis (DKA) within 10 days of the COVID-19 vaccination in three patients. Their cases included two male patients aged 87 and 60 years with known type 2 diabetes histories presented with polyuria, blurry vision, and a dry mouth. Both were diagnosed with HHS-DKA and DKA, with HbA1c levels of 7% and 7.5%, respectively, upon receiving the first dose of the Moderna mRNA-1273 SARS-CoV-2 vaccine. The other case was of a 51-year-old woman who was non-diabetic before and was diagnosed with new-onset T2DM and non-ketotic HHS after a dose of Pfizer-BIONTECH vaccination, along with a history of polyuria, polydipsia, and dizziness within two days after the vaccination. PCR was detected as negative in all cases, along with an increase in inflammatory markers, and the patients recovered rapidly with subcutaneous insulin therapy that was tapered off to oral medication for diabetes [27].

In April 2021, Sculli et al. took a gross overview of the literature on the vaccination of pregnant and breastfeeding women who had diabetes and reported that the COVID-19 vaccination may lead to an increase in humoral immunity response against pancreatic islets, but still, the benefits of vaccination outweigh the risks. Vaccines should be administered to breastfeeding women without taking any pause in breastfeeding, and diabetic women should be vaccinated in the phase of pre-planning pregnancy [28].

A self-controlled case series analysis was conducted in Hong Kong between February 2021 and January 2022 by Yan et al. in order to check the adverse effects of two vaccines, CoronaVAC (Sinovac Biotech Ltd., Beijing, China) and BNT162b2 (Pfizer Manufacturing, Belgium), in type 2 diabetic patients with an age >16 and the incidence of acute complications of diabetes after receiving either one or two doses of vaccines. After using the conditional Poisson regression method and following up for 21 days after vaccination, 141,224 patients who had received BNT162b2 and 209,739 patients who had received CoronaVAC did not show any significant increase in the risk of acute diabetic complications (hypoglycemia, diabetic ketoacidosis, or hyperglycemic syndrome) or other adverse events related to cardiac, metabolic, hormonal, or neurological systems. Thus, it was concluded that the absolute risk increment was likely minimal as compared to the adverse events caused by T2DM during the SARS-CoV-2 disease, given the fact that vaccination outweighs risks or complications in patients with type 2 diabetes [29].

Vaccination and preexisting/new-onset type 1 diabetes mellitus (T1DM) and hyperglycemia

The first case reports of diabetic ketoacidosis development in T1DM after the COVID-19 vaccination were presented in December 2021 by Yakou et al., who reported two female patients known to have T1DM, who developed DKA after the Pfizer-BioNTech vaccination. Among them, a 71-year-old female patient presented with nausea, fatigue, decreased water and dietary intake, and unconsciousness within three days of the Pfizer-BioNTech COVID-19 vaccination. The other was a 52-year-old female patient who developed hyperglycemia and weight loss after the first dose of the Pfizer vaccine and was on insulin aspart after that, until she developed nausea, palpitation, respiratory distress, and severe acidosis immediately after the second dose of the Pfizer vaccine, after which she was managed with intravenous insulin and other therapies, and her DKA recovered gradually [30].

Ganakumar et al. reported two cases of younger adults between the ages of 20 and 25 with the development of diabetic ketoacidosis after vaccination in December 2021 in India. Both were known cases of T1DM for six years, were on regular insulin therapy, and had received their second doses of the inactivated virion-based vaccines COVASHIELD (ChAdOx1 nCoV-19) and COVAXIN (inactivated whole-virion vaccine BBV152; Bharat Biotech, Hyderabad, India) three and six days before symptom aggravation, respectively. The pool of thoughts for the reason behind DKA was the development of oxidative stress caused by the excessive response of protein kinase C to vaccination-induced hyperglycemia, resulting in advanced glycated end products [31].

Zilbermint et al. reported a case of a 24-year-old young adult female with known T1DM who developed rapid-onset DKA with transient profound insulin resistance. She developed severe nausea, tachycardia, tachypnea, and hyperglycemia, leading to ketoacidosis within 15 hours of receiving the second dose of the mRNA Moderna vaccine, which did not resolve with multiple boluses of insulin. She was brought into emergency care, and her DKA was resolved with large insulin infusion therapy for three days [32].

Heald et al. worked on the examination of blood glucose profiles in 96 case reports of T1DM adult patients before and after their first COVID-19 vaccination and reported that the vaccination can lead to hyperglycemia for a few days in some individuals, but can be managed with proper therapy; hence, patients with T1DM should be educated about the side effects before the vaccination implication for the first time [33].

In December 2021, Patrizio et al. reported a case of new-onset of Grave’s disease along with the conversion of T2DM into T1DM in a 52-year-male with known DM for eight years who had good glycemic control and a history of vitiligo vulgaris but no thyroid disease before. He presented with the symptoms of 7 kg weight loss, asthenia, mild thyromegaly without tenderness, and night fever after four weeks of the Pfizer-BioNTech vaccination. His profile showed poor glycemic control with marked hyperglycemia, an increase in HbA1c levels, a decrease in peptide-C, positive organ-specific antibodies, USG characteristics of grave disease, and positive glutamic acid decarboxylase autoantibodies for the first time, making the diagnosis of T1DM and Graves' disease. The patient was treated with insulin to achieve glycemic control and other management [34].

In March 2022, Dicembrini et al. conducted a study on adults with T1DM to determine the safety of the mRNA Moderna vaccine and concluded that vaccination in these patients can lead to the development of generalized reactions of inflammation, including fever, myalgia, nausea, diarrhea, and vomiting, but no such negative effects on glycemic control were noted on both doses of Moderna vaccine, so vaccination can be given in T1DM even if some worrisome events occur [35].

Another case of new-onset T1DM was reported by Yano et al. in 2022. The patient was a 51-year-old pre-diabetic woman with a positive family history of T2DM in her father. The patient got the same symptoms as other cases of polyuria, polydipsia, increased thirst, and weight loss after the first dose of Moderna mRNA-1273 SARS-CoV-2 vaccine (Moderna, Inc., Cambridge, MA), hyperglycemia, and new-onset vaccine-induced autoimmunity, leading to new-onset T1DM. The patient was treated with insulin glargine for glycemic control. Laboratory examinations revealed hyperglycemia with elevated hemoglobin A1c level, metabolic acidosis with an increased anion gap (31.8 mEq/L), and ketonemia, which were consistent with a diagnosis of diabetic ketoacidosis [36].

Safety analysis of mRNA and viral vector-based vaccination

An extensive research study regarding the safety analysis of mRNA vaccination and viral vector-based vaccination was done by di Mauro et al. in the European population. Their study included individual case reports (ICRs) of any adverse or impaired glucose metabolic events (i.e., hyperglycemia, T1DM, diabetes in pregnancy, diabetic emergencies or adverse events, pre-diabetes, and T2DM occurring after vaccination). Their study reported a higher ratio of hyperglycemia and hypoglycemia regardless of diabetes presence in either type of vaccine. Whereas the most common side effects of vaccination were generalized fatigue, chills, fever, and injection site localized pain, mRNA vaccination showed more of the adverse events described above in comparison to vector-based vaccines. However, the events recovered with favorable outcomes within the range of two to three days [37].

Sasaki et al. reported fulminant T1DM for the first time in a 45-year-old Japanese woman who had stable bronchial asthma but no diabetes. The patient got general fatigue and increased thirst with a mild fever after her first dose of the Pfizer-BioNTech vaccine, and within six days, she lost 6 kg of weight along with developing nausea and abdominal pain. Her examination showed hyperglycemia with increased anion gap and acidosis; there were also slightly increased HbA1c levels with the positive islet-associated autoantibodies and serum pancreatic enzymes diagnosing fulminant type I diabetes. The patient recovered with insulin glargine and insulin lispro and was discharged after two weeks [38].

In March 2022, another case of a 50-year-old male patient, with no prior history of diabetes but a positive family history of T2DM in his mother, was presented, with the abrupt development of polyuria and polydipsia six days after the first dose of a COVID-19 vaccine. Immediately after vaccination, the patient got a fever for five days and, the next day, developed hyperglycemia with relatively decreased HbA1c levels, even in the presence of marked hyperglycemia. The case was diagnosed to be new-onset fulminant T1DM and acidosis that was resolved by subcutaneous insulin [39].

Another case of fulminant T1DM, reported by Sakurai et al., was presented in a 36-year-old healthy woman with no personal or family history of diabetes mellitus or autoimmune disease. The patient was presented in emergency care with polyuria, polydipsia, fatigue, loss of appetite, and palpitations three days after receiving the Pfizer-BioNTech vaccination. Her evaluation showed sudden ketoacidosis and marked hyperglycemia, a marked reduction in C-peptide levels and HbA1c, and negative islet-related autoantibodies. She was managed by intravenous fluid replacement and insulin infusion [40].

Kobayashi et al. presented a similar case in June 2022 of a 59-year-old male with no prior personal or family history of diabetes or autoantibody formation. The difference with the other cases was that the patient presented with worsening general fatigue and a few episodes of vomiting within 15 days after receiving the second dose of the mRNA Pfizer-BioNTech vaccination. The investigation showed the same profile as the other cases (i.e., hyperglycemia, low C peptide, etc.). However, with GAD-positive antibodies, the patient was diagnosed with DKA, along with fulminant T1DM. He was treated with intravenous (IV) insulin that was substituted with subcutaneous insulin for glycemic control. The school of thought for the reason was that rapid insulin depletion led to decreased immune response and anti-GAD antibody formation, with HLA analysis showing suspicion of fulminant T1DM [41].

In November 2022, fulminant T1DM development was reported by Ling et al. in a 39-year-old non-diabetic woman with a positive family history of T2DM. She had been vaccinated eight months before with the recombinant spike protein vaccine Medigen, but after receiving a booster dose of the Pfizer-BioNTech vaccine 14 weeks before admission, she developed signs and symptoms that were first misdiagnosed as fresh T2DM on admission from a hospital due to a DKA episode and were relieved by insulin short-term infusion therapy and anti-diabetic medicine for three days admission, but that led to a second DKA episode and worsening signs and symptoms of dyspnea, palpitations, severe nausea, and vomiting several times a day with a temperature 36°C, and admission into emergency in Lin et al.’s hospital. On her examination, there was hyperglycemia and ketoacidosis, as well as high anti-GAD antibodies. She was diagnosed with fulminant T1DM and treated with insulin pump therapy in an emergency and later with a four-split insulin regimen. The patient had to continue insulin boluses for months after discharge. Just like the cases reported by Sasaki et al. and Sakurai et al., the cases of Ling et al. showed positive autoantibodies against pancreatic islet cells (anti-islet antigen 2) [42].

Kshetre et al. reported a case of new-onset T1DM development in a pre-diabetic hypertensive male aged 69 years. The patient got his first dose of the mRNA COVID-19 vaccine one year ago, with a second dose two months before aggravation of symptoms. The patient presented with polyuria, polydipsia, and loss of taste and appetite for two weeks. On investigation, he showed increased HbA1c levels, decreased c-peptide levels, hyperglycemia, elevated anion gap, elevated creatinine, glycosuria, and ketonuria, all diagnosed as DKA and acute kidney injury. His hyperglycemia was managed by insulin infusion in the intensive care unit, and he was discharged with home medication of insulin. This rapid progression of pre-diabetes into T1DM was supposed either to occur through damage to pancreatic cells due to the mRNA vaccination or due to cytokine-mediated beta cell damage [43].

Sasaki et al. reported another case of new-onset T1DM in January 2022 in a 73-year-old female who had a history of well-controlled mild hyperglycemia, with a diet without any anti-diabetic medications and no positive family history of T1DM 1 or T2DM. After four weeks of receiving two doses of the Moderna mRNA vaccine and passing mild phases of fever and malaise at the time of vaccination, the patient got sudden anorexia, nausea, vomiting, and lab reports of high HbA1c, low peptide C, poor glycemic control, and insulin autoantibodies formation, diagnosing it as T1DM and meeting the criteria of autoimmune/auto-inflammatory syndrome induced by adjuvants (ASIA) syndrome. After receiving insulin therapy, the patient’s hyperglycemia was controlled, but the patient became dependent on insulin therapy from then on due to T1DM. The authors suspected mRNA involvement in self-adjuvant properties, thus causing insulin resistance and autoantibody formation [44].

In June 2022, Piccini et al. conducted a study on a younger population of mean age 18 with T1DM to determine the adverse effects of the vaccination in them. All 39 individuals received full-cycle doses of either the Pfizer-BioNTech vaccine or the mRNA Moderna vaccine with glucose monitoring and symptom checking before and after receiving the vaccine. The common signs and symptoms were pain at the injection site, mild fever, headache, and myalgia, but did not exhibit sudden hyperglycemia [45].

Makiguchi et al. presented a case of diabetic ketoacidosis in January 2022. That patient was a non-diabetic female with adenocarcinoma of the lungs with brain metastasis who had undergone seven months of nivolumab and ipilimumab (NIC) therapy with controlled disease. On receiving her first dose of the Pfizer-BioNTech vaccine, she got polyarthralgia and fever, but the second dose led to appetite loss, fatigue, and truncal erythema. Investigations showed DKA, suggesting T1DM. She was discharged with permanent insulin therapy, and the authors suggested that DKA was supposed to be immune-related due to the COVID-19 vaccination during NIC therapy [46].

Effect of hyperglycemia on immunogenicity

The immunogenicity, glucose-related metabolic effects, and safety checks of the mRNA-based COVID-19 vaccine were done by D’Addio et al. in a cohort study involving 326 known cases of T1DM and 49 non-diabetic yet positive T1DM family histories. The effects were observed and compared after both doses of vaccination. Their study showed an impaired or reduced immune response specific to SARS-CoV-2 mRNA with no increase in cytotoxic factors or cytokines as compared to non-diabetic or healthy individuals, whereas there was no effect on humoral immune response or glycemic control after two doses of mRNA vaccination [47].

In a letter to the authors of the Italian Society of Endocrinology, Bleve et al. presented new-onset autoimmune diabetes development in two Caucasian women of age 57 and 61 years after the viral vector-based vaccines AstraZeneca and Pfizer-BioNTech vaccine, with presentation times of seven days and 26 days, respectively. The female receiving the AstraZeneca vaccine presented with typical signs and symptoms of diabetes mellitus (i.e., polyuria, polydipsia, and fatigue); lab investigations showed glycosuria, ketonuria, positive anti-GAD antibodies, anti-tyrosine phosphatase antibodies, and anti-transglutaminase IgA antibodies. Meanwhile, the other female receiving the BioNTech vaccine presented with dyspnea and nausea. Investigations showed high fasting plasma glucose, acidosis, and acute abdomen, along with raised anti-GAD and anti-thyroid peroxidase antibodies. The patients were treated with basal insulin therapy and discharged with insulin prescriptions. The authors further declared that the adverse effects may be due to immune-related events caused by COVID-19 vaccination that are either triggered in already glycemic patients or due to immune responses that cause inflammatory cytokine production, leading to insulin resistance and damage to autoimmune beta-cells of the pancreas, as observed by different studies discussed before [48].

A case of fulminant T1DM in a 43-year-old male with malignant melanoma was presented by Sato et al. in January 2022. The patient was on immune checkpoint inhibitor (ICI) therapy with nivolumab with glycemic levels for the past year. On receiving the first dose of vaccination for SARS-CoV-2, the patient developed localized symptoms, but two days after receiving the second dose, the patient developed typical symptoms of diabetes mellitus with weight loss, investigations showing low C peptide levels, high blood glucose levels, and insulin resistance that were treated first with intravenous insulin and later with intracutaneous insulin after two days. The authors proposed that vaccination leads to fulminant T1DM by targeting already at-risk immune and glucometabolic systems [49].

Another case study of diabetic ketoacidosis in a 77-year-old woman after the second dose was presented by Nishino et al. in April 2022. The patient received first dose of the SARS-CoV-2 vaccine tozinameran (BNT162b2) 23 days before and had multiple previous comorbidities, including obesity, hypothyroidism, breast cancer with left breast surgery, metastatic brain tumor, T2DM that was converted to pembrolizumab-induced fulminant T1DM at the age of 75, and chemotherapy, but there was no history of diabetic ketoacidosis. After two days of administration of the second dose of the vaccine, she developed unconsciousness. Upon laboratory examination, there was hyperglycemia, and a high anionic gap, while COVID-19 PCR was negative. The patient had DKA with the development of right hemiplegia due to multiple infarct formation inside the brain because of stenosis. The patient was admitted to the intensive care unit, mechanically ventilated with an infusion of insulin and normal saline, and got back on the normal GCS with the recovery of DKA and oral food intake on the 11th day of admission. He was discharged one month later. The authors suggested the development of DKA was due to vaccine and drug-induced adverse reactions [50].

Moon et al. reported a case of a 56-year-old woman in January 2023 who developed new-onset autoimmune T1DM after receiving the second dose of the Moderna mRNA vaccine with no personal or family history of autoimmunity but a positive T2DM history in her mother. The patient presented with polyuria, polydipsia, and weight loss. She was managed by oral medications first and then insulin later on. Her profile showed controlled glycemic levels with insulin but decreased C-peptide levels with GAD antibodies that were consecutively positive after discharge. This was possibly the first case of mRNA vaccination in which the patient didn’t develop DKA after mRNA vaccination yet showed T1DM, hence showing that the patient’s C-peptide levels and autoantibodies should be kept in mind even in the absence of DKA after the patient shows symptoms followed by vaccination [51].

In October 2022, a series of case studies were reported by Aydoğan et al. in Japan after the emergence of four cases of T1DM after receiving COVID-19 vaccination from Pfizer-BioNTech. The patients presented with the typical symptoms of dehydration, polyuria, dyspepsia, and poor glycemic control; investigations of these patients showed hyperglycemia in accordance with GAD65Ab autoantibodies against glutamate decarboxylase in three patients, while one patient had autoantibodies against the thyroid. The patient was diagnosed with T1DM, and the authors discovered that impaired glucose regulation was caused by vaccination-induced autoimmunity [52]. Mungmunpuntipantip et al. explaining T1DM following SARS-CoV-2 mRNA vaccination reported that the effects might not be surely due to COVID-19 vaccination directly, but could be due to other comorbidities as well as dengue fever that might lead to T1DM [53].

On the contrary to all of the above, a prospective study was conducted by Aberer et al. in February 2022 to investigate the short-term term effects of COVAC-DM vaccination in preexisting patients with T1DM and T2DM by glycemic and symptom monitoring. Their study on 161 people concluded that COVID-19 vaccination did not show adverse effects on preexisting T1DM and T2DM; instead, deterioration of blood glucose was found at the time of symptoms of headache, fatigue, and myalgia after the vaccination [54].

Prevalence of SARS-CoV-2 in diabetic patients after vaccination

Carrondo et al. observed the prevalence of SARS-CoV-2 in 100 patients of T2DM after receiving COVID-19 vaccination. They concluded that, even after the vaccination the patients are prone to SARS-CoV-2 infection, given the fact that obese patients with T2DM are more prone to it. Moreover, multiple booster doses are required in order to achieve and maintain the efficacy of the immunity [55].

Marfella et al. conducted a study in the Italian population to see the effect of hyperglycemia on the immunogenicity of COVID-19 vaccination and concluded that hyperglycemia can worsen the immune response of the body, whereas a good immune response can be achieved with proper glycemic control following COVID-19 vaccination [56].

The pre-diabetic population is more prone to develop new-onset diabetes type 1, with fulminant type 1 diabetes most evidently occurring after the vaccination. Adults of any age can develop new-onset diabetes, or the exacerbation of diabetes can occur with diabetic ketoacidosis; that is, there is no specific age group that is more prone to develop new-onset diabetes type 1 or diabetic ketoacidosis. Moreover, almost all of the diabetic complications occurring after vaccination showed a sudden rise in blood glucose levels, sudden severe hyperglycemia, low peptide C levels, the formation of anti-islet autoantibodies or GAD65 autoantibodies, and signs of ketoacidosis, including polyuria, polydipsia, fever up to 38 °C, and general fatigue. In some cases, intensive care might be needed along with ventilation. Other signs included respiratory distress and palpitation, mostly with negative COVID-19 PCR. Pfizer-BioNTech and Moderna mRNA vaccinations have been associated with the occurrence of most diabetic complications, whereas CoronaVac appeared to be relatively safe in diabetic patients. The exact causes are still unknown and need to be discovered, but the possible causes proposed are the effects of the mRNA-induced autoantibody formation due to some of its properties that aggravate them. The role of insulin resistance is unclear behind hyperglycemia and diabetic ketoacidosis development after COVID-19 vaccination. Further research is needed to evaluate in depth the mechanisms causing diabetic complications.

## Conclusions

After the presentations of diabetes and new onsets after vaccination, almost all of the patients presenting with either ketoacidosis or fulminant T1DM have recovered and well managed through insulin infusions, achieving good glycemic control, so the benefits of the vaccination outweigh their side effects. Hence, it is suggested to educate and counsel the pre-diabetic or preexisting diabetic population in a community setting about the possible adverse effects of the vaccination and their signs and symptoms, along with educating them for self-management prior to developing hyperglycemia and reporting any complications to the hospital care setting. During the pandemic, there is a need that patients with preexisting diabetics, pre-diabetics, or those populations with a positive family history of diabetes should be monitored carefully and checked up routinely for glycemic control.
